# On Pneumonia Biliosa

**Published:** 1831-01-01

**Authors:** 


### LXIV.

### ON PNEUMONIA BILIOSA. By R. N. ALLEN, M.D. of the United States.

IN our Maryland contemporary, of July last, a paper on the above disease is given from the pen of Dr. Allen. The title of bilious pleurisy, or peripneumony, may sound rather strange to English ears; but there is unquestionable foundation for the appellation, even in this country, as we have often, and even very recently, had occasion to know. In hotter climates, and especially in America, bilious pleurisy is very common, and sometimes epidemic.

"SYMPTOMS :—Commonly, but by no means universally, the invasion of pneumonia biliosa, of whatever grade, is marked by a distinct chill. This is sometimes substituted by slight sensations of chilliness, frequently of considerable duration, and occasionally extending even to several days. Premonitory symptoms sometimes precede the attack, similar to those which precede the occurrence of other febrile diseases, but generally accompanied also by catarrhal affections. It happens, indeed, not unfrequently, that the severest forms of bilious pleurisy supervene on catarrh, by gradual and insensible aggravation of the catarrhal symptoms."

"It occasionally happens, even in the most acute cases, that there is no cough whatever, but in the great majority, it is from the beginning a troublesome symptom. Sometimes it is almost incessant, greatly aggravating the pain, and producing extreme distress.

The expectoration is at first scanty, but becomes more copious as the disease advances. In perhaps about half of the well-marked cases, the sputa are streaked with blood, nor does this symptom at all darken the prospect of recovery. It is observed, however, by Professor Potter, that the appearance of grumous blood in the matters expectorated, is an unfavourable symptom. Our own experience has thrown little light on the science of prognosis. The sputa are also frequently, if not generally, tinged with bile, but this circumstance being common, can afford no ground for any conjecture as to the event. We have not observed the spitting of blood attending pneumonia to render the patients peculiarly liable to chronic diseases of the lungs.

Thoracic pain is an universal symptom, but it occupies various parts of the chest. Sometimes it is confined to a single spot, at other times it is diffused through various parts of the thorax. The breast and sides are its most common seats, but it occasionally invades the back, about the region of the lower ribs, and wherever it may be seated, very frequently extends through the breast to the scapulæ. The pain is, perhaps, universally aggravated by a full inspiration, but this circumstance is by no means diagnostic, as is often supposed. We have sometimes observed the pain attendant on the most transient attacks of colic, as well as that accompanying other abdominal affections, to be also aggravated by inspiration.

Pain of the head attends a certain proportion of cases, perhaps not more than one-third or one-half the whole number. It seems to be purely symptomatic of the gastric or pulmonary disorders, seldom continues beyond the early stages, and presents no distinct indications in the treatment. We have never known it to terminate in phrenitis, nor has it ever required any other remedy besides a dark room, an elevated position of the head, and blisters to the neck. Professor Potter observes that the pain of the head is in inverse proportion to the thoracic pain, but we must confess that we have never seen any thing to warrant such an assertion."

"The state of the respiration we regard as far more important in the prognosis, of pneumonia, than all the other symptoms together. It is impossible, however, for laborious breathing to continue long without seriously affecting the pulse.

The tongue is perhaps uniformly coated with a yellow fur. If the disease should terminate without the supervention of typhoid symptoms, which happens in a great majority of cases, the appearances of this coat will be but little changed during its progress. The cleaning of the tongue at its tip and edges is one of the earliest and most decisive indications of a favourable crisis. Where the typoid condition supervenes, which frequently occurs in protracted cases, it becomes first brown and dry, and afterwards black, or of a smooth shining red, as if covered with an adventitious pellicle. Here again, a change from these appearances towards the natural condition, will afford the most decisive sign of convalescence."

"The dejections are almost uniformly bilious, and for the most part retain this character for several days from the attack. The colour of the skin and eyes, and the serum discharged from blisters, are frequently concurrent signs of the bilious character of the disease."

"Unless typhoid symptoms occur, pneumonia biliosa commonly terminates in from seven to fourteen days. Its duration within those limits is wholly uncertain and indeterminate. If the typhoid condition should supervene, it will generally be protracted for one or two weeks longer."

We lately met with a marked case of this kind in Great Russel Street, which terminated fatally on the 14th or 15th day. The patient was an elderly gentleman, who had served many years in the East Indies, and laboured under hepatic derangement. Having got cold, however, in the beginning of last November, he was seized with unequivocal pleuritis and also pneumonia of the right lung, exhibiting all the usual phenomena. We were rather surprised, however, when the expectoration commenced on the second or third day of the disease, to find it as yellow and as bitter as bile—in short, it consisted in a great measure of bile. About the 5th or 6th day, the skin also began to assume a yellow hue, and all the secretions and excretions partook of the same colour. The expectoration never altered into the bland muco-purulent kind; but continued viscid and bilious till the last. The fever assumed a typhoid type, and the patient sank exhausted at the end of a fortnight. It is exceedingly difficult to account for the yellow colour and bitter taste of the expectoration in cases of this kind.

---

